# An automatic measure for speech intelligibility in dysarthrias—validation across multiple languages and neurological disorders

**DOI:** 10.3389/fdgth.2024.1440986

**Published:** 2024-07-23

**Authors:** Johannes Tröger, Felix Dörr, Louisa Schwed, Nicklas Linz, Alexandra König, Tabea Thies, Michael T. Barbe, Juan Rafael Orozco-Arroyave, Jan Rusz

**Affiliations:** ^1^ ki elements GmbH, Saarbrücken, Germany; ^2^ Cobtek (Cognition-Behaviour-Technology) Lab, University Côte d’azur, Nice, France; ^3^ Centre de Mémoire de Ressources et de Recherche, Centre Hospitalier Universitaire Nice (CHUN), Nice, France; ^4^ Department of Neurology, Faculty of Medicine and University Hospital Cologne, Cologne, Germany; ^5^ IfL Phonetics, Faculty of Arts and Humanities, University of Cologne, Cologne, Germany; ^6^ GITA Lab, Faculty of Engineering, University of Antioquia, Medellín, Colombia; ^7^ Pattern Recognition Lab, Friedrich-Alexander-Universität Erlangen-Nürnberg, Erlangen, Germany; ^8^ Department of Circuit Theory, Czech Technical University in Prague, Prague, Czechia

**Keywords:** amyotrophic lateral sclerosis (ALS), Huntington's disease (HD), Parkinson's disease (PD), progressive supranuclear palsy (PSP), speech analysis, intelligibility, digital biomarkers

## Abstract

**Introduction:**

Dysarthria, a motor speech disorder caused by muscle weakness or paralysis, severely impacts speech intelligibility and quality of life. The condition is prevalent in motor speech disorders such as Parkinson's disease (PD), atypical parkinsonism such as progressive supranuclear palsy (PSP), Huntington's disease (HD), and amyotrophic lateral sclerosis (ALS). Improving intelligibility is not only an outcome that matters to patients but can also play a critical role as an endpoint in clinical research and drug development. This study validates a digital measure for speech intelligibility, the ki: SB-M intelligibility score, across various motor speech disorders and languages following the Digital Medicine Society (DiMe) V3 framework.

**Methods:**

The study used four datasets: healthy controls (HCs) and patients with PD, HD, PSP, and ALS from Czech, Colombian, and German populations. Participants’ speech intelligibility was assessed using the ki: SB-M intelligibility score, which is derived from automatic speech recognition (ASR) systems. Verification with inter-ASR reliability and temporal consistency, analytical validation with correlations to gold standard clinical dysarthria scores in each disease, and clinical validation with group comparisons between HCs and patients were performed.

**Results:**

Verification showed good to excellent inter-rater reliability between ASR systems and fair to good consistency. Analytical validation revealed significant correlations between the SB-M intelligibility score and established clinical measures for speech impairments across all patient groups and languages. Clinical validation demonstrated significant differences in intelligibility scores between pathological groups and healthy controls, indicating the measure's discriminative capability.

**Discussion:**

The ki: SB-M intelligibility score is a reliable, valid, and clinically relevant tool for assessing speech intelligibility in motor speech disorders. It holds promise for improving clinical trials through automated, objective, and scalable assessments. Future studies should explore its utility in monitoring disease progression and therapeutic efficacy as well as add data from further dysarthrias to the validation.

## Introduction

Dysarthria is a motor speech disorder resulting from weakness or paralysis of speech-related muscles (1). It leads to decreased speech intelligibility, frequent communication breakdowns, and a reduced quality of life. Speech intelligibility is reduced in many types of dysarthria, including typical Parkinson's Disease (PD) (2–5), atypical parkinsonism such as progressive supranuclear palsy (PSP) (4, 6, 7), Huntington's disease (HD) (8, 9), amyotrophic lateral sclerosis (ALS) (1, 10), and multiple sclerosis (MS) (11, 12).

**Figure 1:**
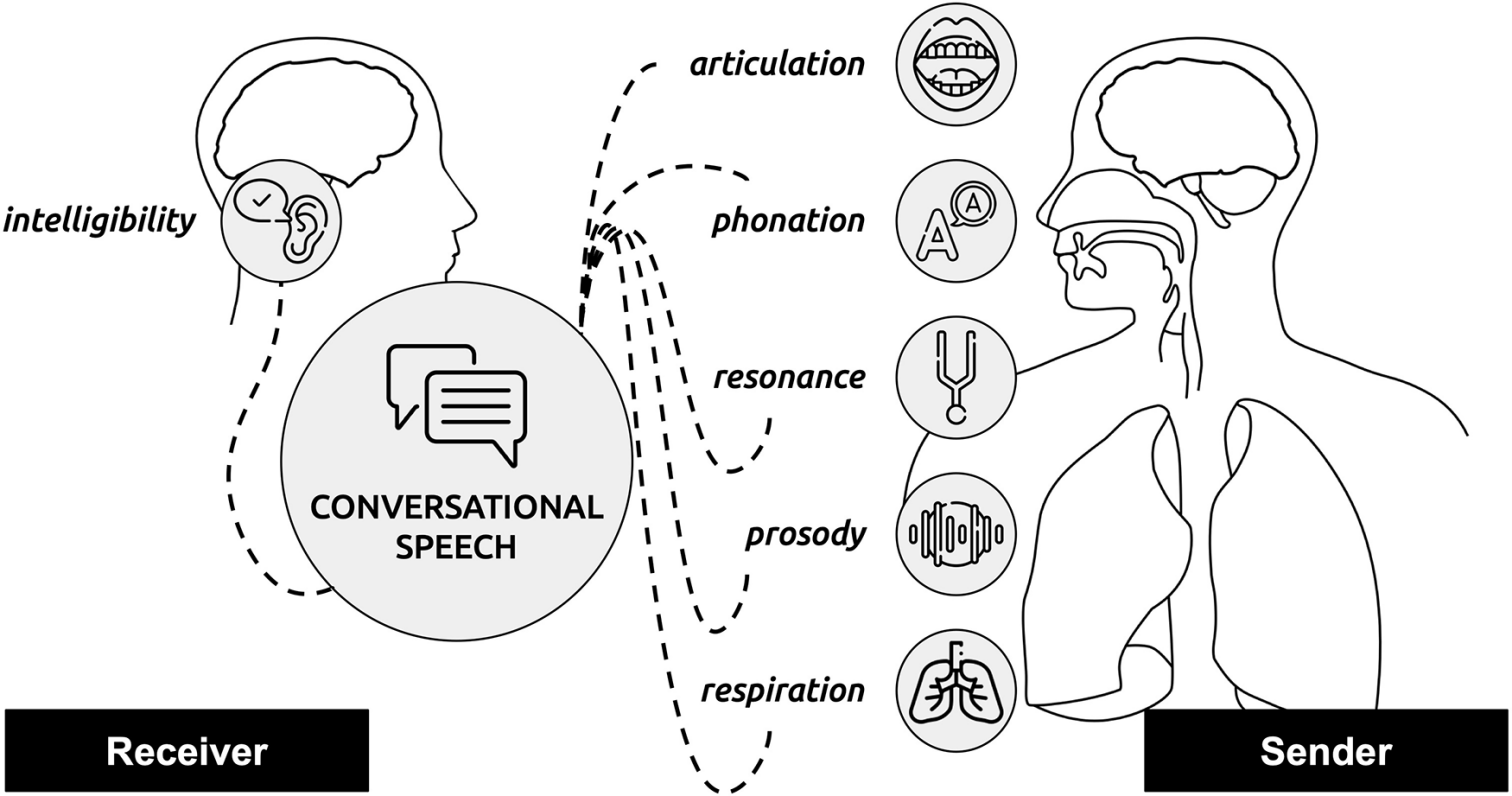
Conceptual model of intelligibility; being a receiver/listener-focused measure and being affected by impaired speech subsystems underlying dysarthrias within the sender: articulation, phonation, resonance, prosody, and respiration.

Reduced intelligibility of patients’ speech often leads to communication difficulties and affects social participation and quality of life in general (13, 14). Hence, communication deficits and perceived intelligibility of their speech represents a major concern for patients with motor speech disorders (15, 16). Speech intelligibility is a construct depending on (a) a speaker (sender) who produces an acoustic signal within, e.g., conversational speech, and (b) a listener (receiver) who receives the signal and interprets it; the success of the interpretation is a direct function of the intelligibility (17) (see also Figure 1). Although a major concern, speech intelligibility is not necessarily dependent on disease severity, duration, or motor phenotype and patients’ own perceptions of the severity do not necessarily reflect objective measures (18). Improved intelligibility is often a primary goal of speech therapy, especially for individuals with dysarthria, and can be a valuable endpoint for clinical research and drug development (19).

Accordingly, measuring speech intelligibility is a clinically relevant assessment for monitoring a dysarthric patient's status and tracking the effectiveness of treatments (20). The common method for assessing speech intelligibility is perceptual evaluation by trained personnel—often clinicians. Standard clinical assessments for disorders associated with dysarthria, such as the Movement Disorder Society Unified Parkinson's Disease Rating Scale (MDS-UPDRS) (21), the Unified Huntington's Disease Rating Scale (UHDRS), and the revised amyotrophic lateral sclerosis functional rating scale (ALSFRS-R) (22), are based on clinician-rated questionnaires and assess, among other symptoms, speech intelligibility. However, these assessments require patient and clinician presence and can be subject to observer bias, pointing to a need for more objective automated methods for assessing speech disorders.

As the field of automated speech analysis is growing in clinical research and healthcare applications, there is increasing potential for digital automatic assessments of speech-related symptoms in motor speech disorders (23, 24). Digital dysarthria assessments are better suited for automated patient-administered screening or stratification at low cost to accelerate clinical trials (24–26). Furthermore, a high level of automation can easily scale up outreach to draw unbiased and representative trial populations beyond established clinical sites and hospital networks. In addition, within clinical trials, digital markers deliver objective high-frequency data to guide interventional clinical trial decision-making and make evaluation more efficient (27).

Previous studies have demonstrated how commercially available automatic speech recognition (ASR) systems could be a feasible platform for automatic measures of intelligibility in patients with motor speech disorders (19, 28). As commercial ASR systems are developed majorly on typical—presumably non-dysarthric—speech, the recognition accuracy of such a system should be an inverse model of the intelligibility of the speaker (29–31).

However, although promising results have been published in feasibility studies, there has not been any comprehensive validation work including multiple pathologies and multiple languages and following a systematic validation framework. The Digital Medicine Society (DiMe) V3 framework (verification, analytical validation, and clinical validation) (32–34) defines validation cases that digital measures should comply with to be considered fit-for-purpose for clinical trials and eventually medical devices, such as digital diagnostics. This framework has gained in importance in recent years and can be regarded as an industry standard for digital measures in this field.

In this study, we present a validation following the DiMe V3 framework for a digital measure for intelligibility, the ki: speech biomarker score for motor speech disorders intelligibility (ki: SB-M intelligibility score). We validate the SB-M intelligibility score in individuals with motor speech disorders, including PD, PSP, HD, and ALS, in multiple languages, including German, Czech, and Colombian Spanish, representing the Germanic, Slavic, and Romance language families.

## Methods

### Data

Four different datasets were used in the analysis: (1) Czech data from *N* = 39 patients with HD (35), *N* = 43 patients with PD (36), *N* = 16 patients with ALS (37), *N* = 17 patients with PSP (6), and *N* = 46 healthy controls (HCs); (2) Colombian data from *N* = 50 HCs and *N* = 50 patients with PD (38); and (3) German data (39) from *N* = 98 patients with PD. For detailed information on the initial cohorts, reading texts, and data collection process, we refer to the initial publications cited; however, for better readability for this manuscript, a short description will be given in the following sections. Compare also Table 1.

**Table 1:** Demographic information of the samples and as essential clinical information.

	German	Colombian		Czech				
	PD DE	PD CO	HCs CO	PD CZ	HD CZ	PSP CZ	ALS CZ	HCs CZ
*N*	98 (32 F)	50 (25 F)	50 (25 F)	43 (19 F)	39 (20 F)	17 (6 F)	16 (11 F)	46 (21 F)
Age (years)	62.7 (±8.23)	61.02(±9.44)	60.98 (±9.46)	63.0 (±9.92)	48.28 (±13.4)	66.76 (±4.8)	60.0 (±10.66)	51.54 (±14.05)
MDS-UPDRS, UHDRS, NNIPPS, ALSFRS-R	37.43 (±10.89)	37.66 (±18.32)	—	20.88 (±10.92)	26.51 (±11.47)	67.12 (±26.7)	35.06 (±6.97)	—
Clinical scale speech items	0.80 (±0.90)	1.34 (±0.82)	—	0.81 (±0.63)	0.81 (±0.46)	1.88 (±0.7)	2.75 (±0.86)	—
ki: SB-M intelligibility score	0.82 (±0.18)	0.73 (±0.18)	0.86 (±0.11)	0.81 (±0.07)	0.67 (±0.17)	0.54 (±0.28)	0.58 (±0.29)	0.85 (±0.04)

CO, Colombian Spanish; CZ, Czech; DE, German.

ALSFRS-R: note that ALSFRS-R has an inverse relationship to disease severity, unlike the other scales where higher scores mean greater severity. Clinical scale speech items: MDS-UPDRS item 3.1, UHDRS dysarthria score, NNIPPS speech item, ALSFRS-R speech item from the bulbar score.

#### Czech data

Participants read an 80-word long paragraph in the respective language, which was phonemically balanced and well-established in clinical research (3). Recordings were conducted in a quiet room with low ambient noise, using a condenser microphone placed approximately 15 cm from the subject's mouth. Each participant had one recording session with the speech-language pathologist, without time limits. Participants were briefed on the speaking tasks and recording process. Each participant provided written informed consent. The collection of the Czech data was approved by the Ethics Committee of the General University Hospital in Prague, Czech Republic (approval number 6/15 Grant GACR VFN).

#### Colombian data

Participants read 10 sentences of increasing complexity (38). Recordings were collected in a soundproof booth at the Clinica Noel in Medellin, Colombia, using a dynamic omnidirectional microphone and a professional audio card. This study was in compliance with the Helsinki Declaration and was approved by the ethics committee of the Clinica Noel in Medellin, Colombia. Written informed consent was signed by each participant.

#### German data

Participants read an 80-word long paragraph in the respective language, which was phonemically balanced, well-established, and taken from the German protocol version of the Dysarthria Analyzer (40). Speech data were collected in the Department of Neurology of the University Hospital Cologne in a room with low ambient noise using a condenser microphone headset to keep the mouth-to-microphone distance constant at approximately 7 cm from the mouth. Each participant provided written informed consent. The data collection was approved by the local ethics committee (protocol code: 23-1461-retro).

After the reading task, patients in all three cohorts underwent a range of clinical assessments (different for each study and cohort), of which the following are important for this study: the MDS-UPDRS (21), UHDRS (41), Natural History and Neuroprotection in Parkinson Plus Syndromes—Parkinson Plus Scale (NNIPPS) (42), and ALSFRS-R (22).

### Automatic speech recognition and intelligibility score

To calculate the automatic intelligibility scores, we first ran the audios from the reading passage and reading sentences (in Colombian Spanish) through SIGMA the ki: proprietary speech processing library, which—besides other preprocessing and feature extraction steps—also interfaces with commercially available ASR systems; for verification, we selected two different providers: Google Speech API (43) and Amazon Transcribe (44). Based on the transcripts and the target reading texts, we calculated the word error rate (WER, error between the number of target words in the reading text and that in the ASR transcripts) and word accuracy (WA, similar to [28]). From those raw measures, we then derived an automatic proxy for the intelligibility of the speech—the ki: SB-M intelligibility score.

### V3 framework

The V3 framework established by the DiMe Society (32) provides a unified evaluation framework for digital measures. V3 includes three distinct phases in sequential order: verification, analytical validation, and clinical validation. For all the three phases, different data have to be collected and statistically analyzed to provide the necessary results.

#### Verification

Verification entails the systematic evaluation of sample-level sensor outputs against prespecified criteria. The ki: SB-M intelligibility score relies on ASR. Therefore, the most critical part of the sensor output and preprocessing pipeline is the automatic transcription of speech. The ki: SB-M intelligibility score uses a proprietary speech processing pipeline leveraging commercial ASR providers. To verify the performance at this stage, we calculated intraclass correlation coefficients (ICCs) for the WER and SB-M intelligibility score between Google and Amazon ASR. Previous studies and our own work have shown that error rates on a low level, such as phoneme error rate, do not necessarily model losses of perceptual intelligibility (45). We performed verification across the whole data sets except for the German PD data due to a lack of consent from patients.

In addition, we computed ICCs between repeated tests for data sets in which participants performed two repeated reading passages (all CZ data sets). Although tests are executed in quick succession, this can provide first insights into the retest reliability of the measures. Based on the current state of the art in the field, we considered an ICC of 0.40 (fair correlation) acceptable for verification (46).

#### Analytical validation

Analytical validation evaluates performance to measure a certain concept of interest (similar to construct validity). The ki: SB-M intelligibility score is related to speech impairments resulting in reduced speech intelligibility. For the analytical validation, we compared the ki: SB-M intelligibility score against established clinical anchor measures for speech impairments or dysarthria in the respective populations. Depending on the pathology, these measures differ: PD → MDS-UPDRS → speech item, HD → UHDRS → dysarthria item, PSP → NNIPPS → speech item, and ALS → ALSFRS-R → speech item (please note that in direct comparison with the other clinical scales, the ALSFRS-R has an inverse relationship to disease severity, meaning patients lose points as the disease progresses). For the comparison with the clinical anchors, we computed Spearman's rank correlation coefficient between the ki: SB-M intelligibility score and the respective speech impairment measure.

#### Clinical validation

Clinical validation evaluates the ability to validly measure clinically meaningful change within an intended scenario, including a specified clinical population. The ki: SB-M intelligibility score is built to measure clinically meaningful change in the intelligibility of speech in dysarthrias. To cover a significant range of dysarthrias, we included clinical validation on the following pathologies: PD, HD, PSP, and ALS.

We performed Kruskal–Wallis test group comparisons in the ki: SB-M intelligibility score between the different diagnostic groups (HC vs. pathology). In addition, we analyzed Spearman's rank correlation between the ki: SB-M intelligibility score and the respective global clinical staging measure: MDS-UPDRS, UHDRS, NNIPPS, and ALSFRS-R.

## Results

### Verification

For verification of the SB-M intelligibility score, we report reliability between the SB-M intelligibility score based on two different ASR methods and reliability between successive performances of the reading task and calculation of the SB-M intelligibility score.

#### Inter-rater reliability for ASRs

We compared different ASRs (Google and Amazon) as the basis for the SB-M intelligibility score. For most of the pathological groups, the ICC between both ASR methods showed a good to excellent performance (ICC equal or above 0.30). However, for Colombian PD data, the ICC was only fair and for Czech PD poor; both were still highly significant. The overall HC ICC (across all languages) was also only poor. For details, compare Table 2. WERs showed similar trends to the final intelligibility score, with the following pattern: HCs < PD < HC, PSP = ALS.

**Table 2:** Agreement between two different ASR methods—Google Speech API and Amazon Transcribe—and the resulting SB-M intelligibility score and raw word error rate.

	HC overall	HC CZ	HC CO	PD CO	PD CZ	HD CZ	PSP CZ	ALS CZ
Google SB-M intelligibility score	0.862 (0.182)	0.853 (0.039)	0.859 (0.200)	0.733 (0.273)	0.810 (0.073)	0.675 (0.173)	0.537 (0.281)	0.590 (0.283)
Amazon SB-M intelligibility score	0.968 (0.088)	0.900 (0.041)	0.980 (0.090)	0.917 (0.177)	0.882 (0.050)	0.775 (0.126)	0.666 (0.28)	0.714 (0.238)
ICC SB-M intelligibility score	0.295 (0.0)	0.180 (0.008)	0.283 (0.0)	0.486 (0.0)	0.290 (0.0)	0.702 (0.0)	0.841 (0.0)	0.869 (0.0)
Google word error rate	0.167 (0.184)	0.238 (0.038)	0.160 (0.2)	0.303 (0.276)	0.288 (0.084)	0.437 (0.154)	0.540 (0.231)	0.479 (0.237)
Amazon word error rate	0.058 (0.113)	0.198 (0.042)	0.032 (0.106)	0.121 (0.202)	0.22 (0.066)	0.372 (0.143)	0.425 (0.228)	0.364 (0.193)
ICC consistency	—	**—**	**—**	**—**	0.75	0.858	0.955	0.982

CO, Colombian Spanish; CZ, Czech.

#### Consistency

Consistency over a short period of time (i.e., the same day in the same assessment reading the paragraph twice) was calculated based on repeated paragraph reading in all groups except the Colombian group, which read multiple sentences of increasing difficulty and not one overall homogenous paragraph. The ICCs for consistency were above 0.70, representing a good to excellent agreement. Compare also Table 2.

### Analytical validation

**Figure 2:**
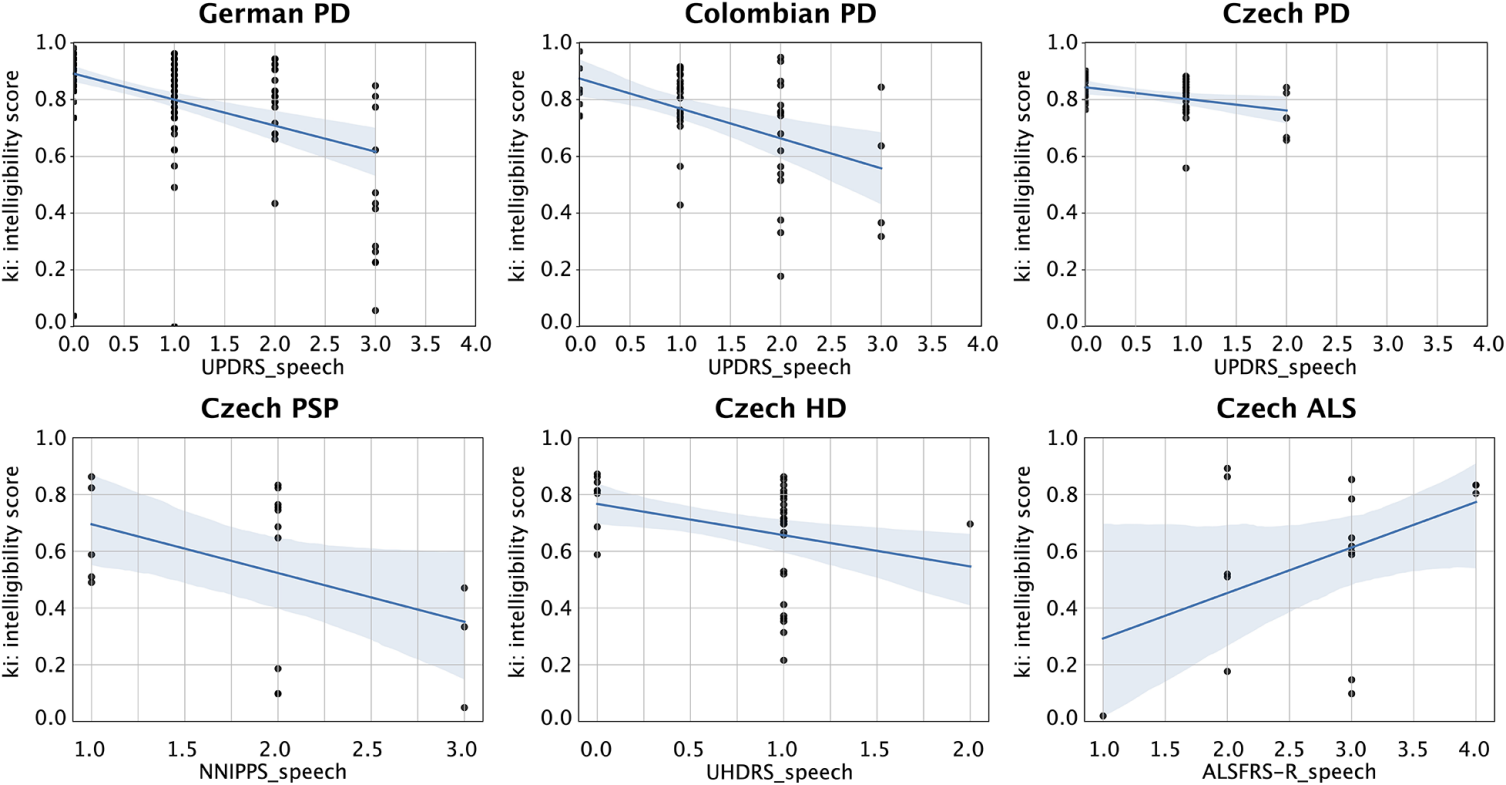
Scatter plots for the correlations between the intelligibility score and respective speech dysarthria clinical anchor score. From upper left to lower right: DE PD correlation with the MDS-UPDRS speech item; CO PD correlation with the MDS-UPDRS speech item; CZ PD correlation with the MDS-UPDRS speech item; CZ PSP DE PD correlation with the NNIPPS speech item; CZ HD correlation with the UHDRS dysarthria score; and CZ ALS correlation with the ALSFRS-R speech item (note that ALSFRS-R has an inverse relationship to disease severity, unlike the other scales in which higher scores mean greater severity). DE, German; CO, Colombian Spanish; CZ, Czech.

For the analytical validation, we compared the ki: SB-M intelligibility score against established clinical anchor measures for speech impairments or dysarthria in the respective populations. We found significant correlations between the intelligibility score and the respective dysarthria anchor score for DE PD (*r* = −0.46, *p* < 0.01, *d* = 1.03), CO PD (*r* = −0.39, *p* < 0.01, *d* = 0.85), CZ PD (*r* = −0.32, *p* < 0.05, *d* = 0.67), and CZ HD (*r* = −0.37, *p* < 0.05, *d* = 0.80). Probably owing to the small sample size, statistically we only found a trend in CZ PSP (*r* = −0.42, *p* < 0.10, *d* = 0.92) and CZ ALS (*r* = 0.32, *p* = 0.21, *d* = 0.68), although effect sizes were medium to large. Compare also Figure 2.

### Clinical validation

**Figure 3:**
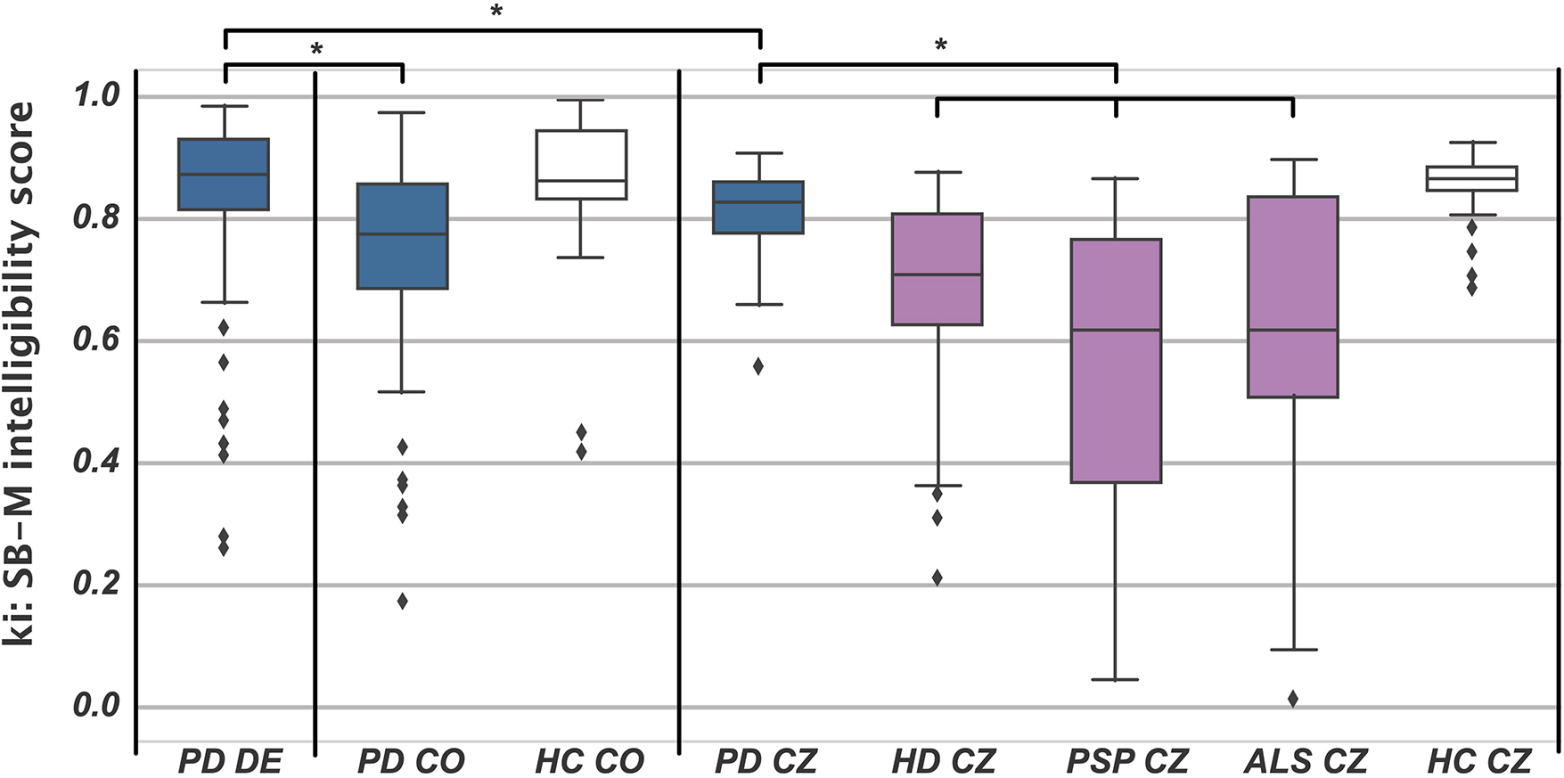
Boxplots of the SB-M intelligibility score for all groups. Blue, PD; white, HC; purple, HD, PSP, and ALS. Asterisks denote a significant *post hoc* group comparison. CO, Colombian Spanish; CZ, Czech.

For the group comparisons, we found significant differences, with the ki: SB-M intelligibility score being significantly lower for the respective pathological group for all cohorts: HC CO > PD CO (*H* = 17.425, *p* < 0.001, *η*^2^ = 0.17), HC CZ > PD CZ (*H* = 13.304, *p* < 0.001, *η*^2^ = 0.14), CZ HC > CZ HD (*H* = 44.437, *p* < 0.001, *η*^2^ = 0.52), CZ HC > CZ PSP (*H* = 29.696, *p* < 0.001, *η*^2^ = 0.46), and CZ HC > CZ ALS (*H* = 18.565, *p* < 0.001, *η*^2^ = 0.29). For description, please see Table 2, and a graphical overview of the group differences is provided in Figure 3.

*Post hoc* group comparisons revealed that the intelligibility scores were comparable for the CZ HD, PSP, and ALS groups, and the CZ PD and CO PD groups. However, German PD showed significantly better intelligibility than the other patient groups, actually performing on a par with the other language HC groups.

## Discussion

This study aimed to validate the ki: speech biomarker for motor speech disorders intelligibility score (ki: SB-M intelligibility score) using the DiMe V3 framework, covering verification, analytical validation, and clinical validation across multiple languages and dysarthria pathologies. Making use of off-the-shelf ASR systems, we took a state-of-the-art approach to automatically measure speech intelligibility in dysarthrias (19, 28, 47). On a conceptual level, we went beyond the aforementioned studies, as we followed the DiME society V3 framework for assessing the readiness of digital measures for clinical research and also included multiple pathologies from the dysarthria spectrum as well as two different ASR systems.

We ran verification on the SB-M intelligibility score, calculating it based on two different automatic speech recognition systems: Google Speech API and Amazon Transcribe. Overall, the ICC indicated good to excellent agreement between the two ASR systems for most pathological groups. However, discrepancies were noted in the Colombian PD and Czech PD data, in which the ICC was only fair to poor, respectively. Poor stability of ASR-based intelligibility measures has been reported previously, especially for typical and mildly impaired severity groups, specifically decreasing their ability to measure changes in the early phases of motor speech disorders (19). The discrepancy might be due to the rather small variance and very good speech recognition, performing almost at an HC level of 0.80, whereas HD, PSP, and ALS have intelligibility scores of 0.70–0.50, with much bigger variances. In these cases, we assume that already small word-level differences inflate discrepancies between ASRs and might cause low ICCs. Especially with the advent of ever-improving ASRs, which also push the needle in dysarthric speech recognition alongside other underrepresented groups, this issue has to be watched closely.

The validity of Google and Amazon ASRs as commercial products naturally extends beyond pathological groups. Both ASR systems have shown high accuracy in recognizing speech from healthy individuals, providing a strong benchmark for comparison (48). However, ensuring robust performance for underrepresented groups remains crucial for the broad applicability and reliability of ASR systems in clinical and everyday settings. On the level of ASR performance in dysarthric speakers, our results compare well with other studies in the field. Gutz et al. (19) found WERs of 10% for mild ALS-related dysarthria to approximately 50% for moderate cases and approximately 80% for severe cases. This is in line with our results for the Czech ALS population, which can be classified as moderately dysarthric based on the ALSFRS-R speech item and shows a 40%–50% WER depending on the ASR system.

Consistency was assessed by comparing the intelligibility scores obtained from repeated paragraph readings. Overall, the ICC values indicated good to excellent consistency. This is an encouraging result but has to be further investigated for repeated measurements of the SB-M intelligibility score assessed longer timeframes apart, such as a couple of days or weeks.

Analytical validation compared the SB-M intelligibility score against established clinical anchor measures for dysarthrias derived from the respective gold standard clinical staging scale. Significant correlations were observed between the SB-M intelligibility score and the respective dysarthria anchor scores for the German PD, Colombian PD, Czech PD, and Czech HD groups. Although specific items are not designed as stand-alone assessments of dysarthria and even less as assessments of intelligibility in principle, we could still demonstrate correlations between the ki: SB-M intelligibility score and those measures. These findings support the SB-M intelligibility score's validity as a measure of perceived speech intelligibility being associated with dysarthria on the speaker side, as confirmed by traditional clinical assessments such as the MDS-UPDRS, NNIPPS, and ALSFRS-R speech items or the UHDRS dysarthria score. Despite medium to large effect sizes, statistical significance was not achieved for the Czech PSP and Czech ALS groups, likely due to smaller sample sizes. Future studies should aim to include larger cohorts to increase statistical power and provide more robust analytical validation.

Our approach to measuring speech intelligibility differs from other research by using a direct measure based on ASR performance, rather than classifying speech into different states/classes of intelligibility. This research is sometimes carried out using machine learning techniques (49, 50). This line of research frames intelligibility as a classification problem, requiring labeled training data to categorize speech into predefined stages. By contrast, our method leverages the continuous output of ASR systems as a proxy for intelligibility, offering multiple benefits. This continuous measure might provide finer granularity and sensitivity to subtle changes in speech quality over time or between groups. In addition, using an off-the-shelf ASR approach eliminates the need for additional machine learning training, making it more accessible and easier to implement in various clinical and research settings.

One of the major limitations of the analytical validation we performed is that we cannot prove this further by comparing with manual intelligibility ratings by either trained professionals or human raters in general, as has been carried out by Gutz et al. in ALS (19). Future studies should add this piece of analytical validation, leveraging existing methods to rate intelligibility by multiple trained and/or untrained raters (51). In addition, our approach presents, in some respect, a black box approach that directly evaluates dysarthria based on intelligibility as perceived by a somehow non-transparent ASR black box. There is a whole research tradition on using carefully crafted acoustic features to estimate dysarthria and different subsystems, as mentioned in the introduction. Pursuing a hybrid approach that taps into ASR-based intelligibility and traditional acoustic analysis features (e.g., pause rate, articulation rate, pitch instability, or monotonicity) to evaluate patients’ dysarthrias would increase the impact of such research and be an important next step.

Clinical validation demonstrated significant differences in SB-M intelligibility scores between healthy controls and pathological groups across all cohorts. This finding underscores the potential of the SB-M intelligibility score as a discriminative tool for identifying and quantifying speech impairments in individuals with motor speech disorders. The consistent pattern of lower intelligibility scores in pathological groups compared with healthy controls across different languages and disorders further supports the robustness and generalizability of the measure. Nevertheless, the experiments presented here still only cover a fraction of the total spectrum of motor-speech-disorder-related dysarthrias or dysarthrias in general. However, our data set of more than 250 patients across four different pathologies and three languages covers a significant amount in this field of research; for rare diseases such as ALS or atypical PD in particular, datasets of that size are rarely reported. In addition, we acknowledge that we did not perform specific testing for cognitive involvement, as the primary aim was to investigate motor speech deviations that are the main contributors to reduced intelligibility. Furthermore, we did not measure the vital capacity of our patients; cohorts such as ALS and PSP may have respiratory impairments that could significantly contribute to reduced intelligibility.

In general, we observed better speech intelligibility in patients with PD than in patients with HD, PSP, or ALS. One reason could be that in the earlier stages of PD, articulation impairment is not as pronounced, allowing for relatively clearer speech. Conversely, HD is characterized by hyperkinetic irregular articulation, and ALS and PSP are associated with hypertonia, leading to imprecise consonant production (52). These speech deficits in HD, ALS, and PSP significantly contribute to reduced intelligibility. These imprecise consonant and uncontrolled (sometimes spastic) irregularities in speech are known to hamper speech intelligibility a lot more than monopitch and monoloudness, which are typically observed in early PD. In addition, the spread in intelligibility scores was a lot greater for HD, PSP, and ALS than for PD, which was also in line with studies on those diseases showing more heterogeneity in their behavioral and speech impairment phenotype.

Between the separate PD groups (DE, CO, and CZ), we observed comparable intelligibility scores in CO and CZ but the German PD group was significantly more intelligible—actually performing on a par with the other language HC groups. This could be related to different recording setups in each study or a general language difference in the underlying ASR performance.

ASR and the measures derived from it exhibit considerable variability when applied to different types of dysarthria (53). Articulatory precision has been identified as the most critical factor influencing speech intelligibility, surpassing the impact of prosody (54).

Finally, another limitation to this study is that we compared intelligibility for audios collected from different studies with different audio recording settings. Although all studies used state-of-the-art microphones for audio recording and professional recording setups—as recommended by recent guidelines (5)—differences in audio recording setups can always play a role in head-to-head comparisons; this is especially the case when comparing our results from CZ directly with CO and DE. Eventually, the accuracy of an automatic speech intelligibility measure is highly dependent on recording conditions. Poor recording environments, such as those with high background noise or subpar microphone quality, can introduce significant bias, leading to artificially low intelligibility ratings. This may result in the erroneous classification of normal speech as dysarthric. Furthermore, different recording devices and handling methods introduce substantial variance, which can confound the measurements and reduce their sensitivity to detect small changes over time or differences between low dysarthria groups. However, one of the most promising scenarios in which to deploy this kind of technology is in at-home environments, where the patient is monitored in everyday life, always using the same device and with similar acoustic conditions. This approach has shown promising results (55). Future studies in this field should adhere even closer to a standardized recording setup or record with multiple devices—one being a standardized microphone setup next to others.

## Conclusion

Overall, this study provides a comprehensive validation of the ki: SB-M intelligibility score for assessing speech intelligibility in motor speech disorders across multiple languages and pathologies. The findings support its reliability, validity, and clinical relevance, highlighting its potential as a standardized tool for clinical and research applications. Automated objective measures of speech intelligibility, such as the SB-M intelligibility score, can increase the efficiency and accuracy of dysarthria assessments, reduce observer bias, and facilitate remote monitoring. This is particularly advantageous for large-scale international clinical trials, in which high-frequency data collection and scalability are critical.

Future efforts should complement validation by investigating the SB-M intelligibility score's ability to monitor disease progression and treatment efficacy. Longitudinal studies assessing changes in the intelligibility score over time and in response to therapeutic interventions could provide valuable insights into the clinical utility of this digital measure.

## Data Availability

The data analyzed in this study are subject to the following licenses/restrictions: the speech data can be accessed from the respective cohort-associated author upon reasonable request. Please navigate through the linked reference for each study in the Methods section. Requests to access these datasets should be directed to rafael.orozco@udea.edu.co, rusz.mz@gmail.com, and tabea.thies@uk-koeln.de.
