# Corrigendum: An automatic measure for speech intelligibility in dysarthrias—validation across multiple languages and neurological disorders

**DOI:** 10.3389/fdgth.2024.1488178

**Published:** 2024-11-13

**Authors:** Johannes Tröger, Felix Dörr, Louisa Schwed, Nicklas Linz, Alexandra König, Tabea Thies, Michael T. Barbe, Juan Rafael Orozco-Arroyave, Jan Rusz

**Affiliations:** ^1^ ki elements GmbH, Saarbrücken, Germany; ^2^ Cobtek (Cognition-Behaviour-Technology) Lab, University Côte d’azur, Nice, France; ^3^ Centre de Mémoire de Ressources et de Recherche, Centre Hospitalier Universitaire Nice (CHUN), Nice, France; ^4^ Department of Neurology, Faculty of Medicine and University Hospital Cologne, Cologne, Germany; ^5^ IfL Phonetics, Faculty of Arts and Humanities, University of Cologne, Cologne, Germany; ^6^ GITA Lab, Faculty of Engineering, University of Antioquia, Medellín, Colombia; ^7^ Pattern Recognition Lab, Friedrich-Alexander-Universität Erlangen-Nürnberg, Erlangen, Germany; ^8^ Department of Circuit Theory, Czech Technical University in Prague, Prague, Czechia

**Keywords:** amyotrophic lateral sclerosis (ALS), Huntington’s disease (HD), Parkinson’s disease (PD), progressive supranuclear palsy (PSP), speech analysis, intelligibility, digital biomarkers

A Corrigendum on An automatic measure for speech intelligibility in dysarthrias—validation across multiple languages and neurological disorders By Tröger J, Dörr F, Schwed L, Linz N, König A, Thies T, Barbe MT, Orozco-Arroyave JR and Rusz J (2024). ***Front Digit Health* 6: 1440986**. **doi:**
10.3389/fdgth.2024.1440986

In the published article, there was an error in the author list, and author Michael T. Barbe was erroneously excluded. The corrected author list appears below.

Johannes Tröger^1^^*^, Felix Dörr^1^, Louisa Schwed^1^, Nicklas Linz^1^, Alexandra König^1,2,3^, Tabea Thies^4,5^, Michael T. Barbe^4^, Juan Rafael Orozco-Arroyave^6,7^ and Jan Rusz^8^

Due to this error the **Author contributions** section has also been updated and the corrected statement appears below.

